# P-2133. Tuberculosis in patients with hematological disease

**DOI:** 10.1093/ofid/ofaf695.2297

**Published:** 2026-01-11

**Authors:** Isabel Ramirez, Pablo Villa

**Affiliations:** Hospital Pablo Tobon Uribe, Universidad de Antioquia, Medellin, Antioquia, Colombia; Hospital Pablo Tobon Uribe, Medellin, Antioquia, Colombia

## Abstract

**Background:**

*Mycobacterium tuberculosis* infection in patients with hematologic disease and hematopoietic stem cell transplant (HSCT) recipients represents a serious complication.

**Figure:**
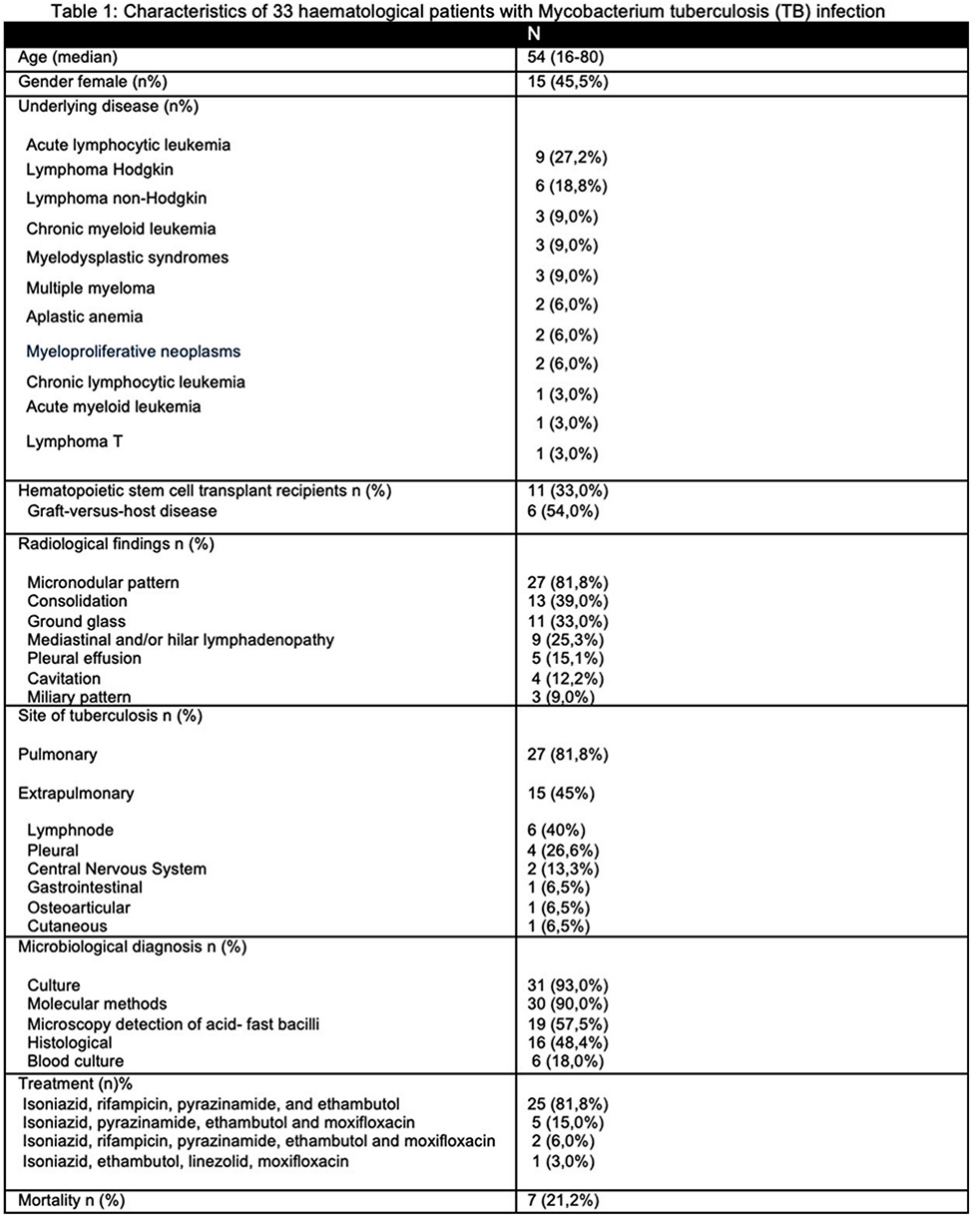

**Methods:**

A retrospective descriptive study was conducted in a high-complexity hospital in Medellín, Colombia, between January 2010 and December 2024. Adults with hematologic disease and a diagnosis of tuberculosis (TB) were included. Sociodemographic, clinical, microbiological, and therapeutic variables, as well as hospital stay and outcome were recorded. Frequency tables were analyzed for qualitative variables, and interquartile ranges for quantitative variables with non-normal distributions, and the mean with standard deviation for normally distributed variables. Data were analyzed using Jamovi 2.3.18 statistical software.

**Results:**

During the study period, 10,307 patients were treated in the hematology service, of which 33 were diagnosed with tuberculosis, with a cumulative incidence of 0.32 %. The most common hematologic disease was acute lymphoblastic leukemia (27.3%). Eleven patients (33%) received HSCT. The median time to onset of TB was 347 days after diagnosis of the hematologic disease, and in patients with a history of HSCT the median time to onset was 300 days.

Pulmonary involvement was the main presentation (81.8%). Extrapulmonary manifestations occurred in 45% of cases, with lymphadenitis (40%) and pleural involvement (26%) being the most common.

Ninety-three percent of cases had positive cultures, 90.9% had a positive molecular test, and 18% had mycobacteremia. The growth time of mycobacteria in culture media was 14 days (5–35 days). Of the patients who received treatment, 21.2% developed drug toxicity. Seven patients died at the end of their hospitalization, resulting in a 21.2% in-hospital mortality rate.

**Conclusion:**

Tuberculosis in patients with hematologic diseases has a low incidence, even in endemic countries. It occurs during chemotherapy or in late stages after HSCT, resulting in significant morbidity and mortality.

**Disclosures:**

All Authors: No reported disclosures

